# *Forward Mentoring*: A Reverse Mentoring-Inspired Initiative to Enhance Cultural Humility and Promote Academic Leadership Capacity

**DOI:** 10.1007/s40670-025-02395-8

**Published:** 2025-04-30

**Authors:** Mali D. Doles, Aneesha Dasgupta, Amy Ribera, Jason D. Doles

**Affiliations:** 1 Higher Education and Student Affairs (HESA), Indiana University, Bloomington, IN 47405 USA; 2 Faculty Affairs and Professional Development (FAPD), Indiana University, Indianapolis, IN 46202 USA; 3 Department of Anatomy, Cell Biology, and Physiology, Indiana University School of Medicine, Indianapolis, IN 46202 USA; 4 Indiana Center for Musculoskeletal Health, Indiana University, Indianapolis, IN 46202 USA

**Keywords:** Mentoring, Leadership, Belonging, Connection, Cultural competency, Professional development

## Abstract

Building relationships and broadening perspectives promotes cultural competence, inclusion, and belonging. At Indiana University School of Medicine, we created an innovative reverse mentoring-inspired program (*Forward Mentoring*) to connect individuals across academic hierarchies with the goal of fostering relationships, cultural humility, and leadership capacity through honest, personal, and reflective discourse—all to build more inclusive and welcoming workplaces. Here we describe the novel *Forward Mentoring* framework, outline the initial programmatic design and implementation, and present an evaluation plan that assesses learning, growth, satisfaction, and program efficacy. Evaluation data from a limited pilot is presented and future directions discussed.

## Introduction

Embracing diversity through inclusive practices increases the breadth and depth of scientific inquiry thus accelerating creative problem-solving. While still a work in progress at many academic institutions, significant investments in pathway programs, recruitment, and retention have yielded steady improvements in representational diversity [1, 29]. Despite these improvements, many institutions have failed to cultivate inclusive environments, especially from the vantage point of individuals who identify with groups that have been historically excluded from the academic research workforce [35]. Alongside other factors, this failure has contributed to high attrition rates among historically marginalized individuals (HMIs) and directly antagonizes other diversity efforts, including recruitment-based interventions [27].

The importance of inclusivity cannot be understated. Researchers have long noted that well-being, belonging, and inclusion track closely with academic thriving. Numerous studies show clear positive associations between perceptions of inclusivity and higher academic performance, persistence, and stronger mental health [22, 31, 39, 40]. A major barrier to inclusivity in academic research is the inherently hierarchical structure of the mentor/mentee relationship. Notable efforts such as those originating from the CIMER Project (Center for the Improvement of Mentored Experiences in Research; University of Wisconsin-Madison) aim to challenge this hierarchy by promoting cultural change and innovative best practices to positively benefit both mentees and mentors [7, 8]. That said, many of these initiatives direct training and development activities to isolated target groups (limiting hierarchy spanning interactions), require significant time investments (usually over a narrow/fixed time window), or lack sufficient follow-up. Indeed, there are not many opportunities to reinforce (or spaces to practice) what is learned at these and other mentoring trainings. We propose that reverse mentoring relationships can be one such space where both mentees and mentors can share openly, voice perspectives, practice communication skills, and ultimately learn from each other [32].

Indiana University School of Medicine is committed to cultivating inclusive educational, research, and clinical environments. Institutional efforts include regular climate assessments (e.g., Diversity Engagement Survey (DES), Professional Fulfillment Index (PFI)) [30, 34], engagement with national consortia focused on well-being and inclusion (e.g., Healthcare Professional Well-being Academic Consortium (PWAC)), and institutional support for inclusion-based activities at multiple levels ranging from individual work unit to all-school efforts. Additionally, there are numerous mentoring programs serving individuals at multiple career stages—the vast majority falling into one of two categories: traditional or peer mentoring. With traditional mentoring, hierarchies are reinforced, and information typically flows unidirectionally and serves to pass on academic or professional advice as well as to support career and personal development [15, 24]. Peer mentoring features a more balanced power structure that can be highly effective, particularly in the social/emotional space where shared life/career-stage challenges can be addressed (and opportunities pursued) in an environment where hierarchy-associated barriers are significantly reduced [14, 17]. In contrast, the basic premise of reverse mentoring is hierarchy challenging role reversal in an experiential learning environment. Here, semi-structured and intimate interactions support motivation and competency building—ultimately enhancing perceptions of belonging and inclusivity for both parties [2].

Despite the growing popularity of reverse mentoring, there remain relatively few examples of its use in academic settings [9, 13]. In the business world, reverse mentoring programs are often designed to address specific needs and frequently entail a junior/newly hired employee serving as a mentor to a senior employee/leader [11]. Early goals of many reverse mentoring programs were to “modernize” senior leadership with respect to technological skills, social media savvy, etc. Recent efforts, including a reverse mentoring program initiated by Eli Lilly, have leveraged this approach to address social justice in the workplace—in their case, building leadership capacity and competency around LGBTQIA + issues. Regardless of the specific context/objective, reverse mentoring seeks to upend traditional power dynamics and hold space for meaningful conversations where both parties have the opportunity to express/share their viewpoints, be heard, and work together on their individual journeys of personal growth and change [23].

*Forward Mentoring* was conceived as a reverse mentoring-inspired professional development activity to promote connection, inclusivity, and cultural humility in academic research environments through perspective broadening conversations and reflection. While not meant to replace efforts to physically diversify faculty/leadership ranks, we contend that programs such as *Forward Mentoring* are essential as they build community and elevate the voices of those who are typically not heard, thus inviting diverse perspectives to the leadership table. Here, we outline our conceptual framework for *Forward Mentoring*, describe key programmatic features, present a summary and evaluation of a limited pilot, and discuss future directions for this work.

## Program Overview

### Approach

*Forward Mentoring* uses experiential learning wherein mentors and mentees are invited to engage with session materials both individually and collectively as they progress through Kolb’s stages of learning, moving from concrete to abstract to active experimentation [26]. Themed-based resources are provided in advance of the reverse mentoring sessions for self-directed study. The *Forward Mentoring* approach combines Kolb’s experiential learning theory and Boyatzis’ self-directed learning model by centralizing relationships, prioritizing reflection and self-awareness, and providing participant-driven individual and collaborative learning opportunities to facilitate personal growth [21, 26]. During the sessions, mentoring pairs engage in dialogue with optional use of themed-based session guides to focus the conversation. These types of mentoring conversations reinforce and embed learning as they allow mentors and mentees to practice the skills that they learn through the resources and guides [38]. The aim is to foster relationships that are constructive versus instructive. This unique approach to reverse mentoring seeks to shift mentoring relationships from a dynamic of “power over” to “power with” to drive transformative change.

### Cohort Selection and Matching

#### Recruitment

The initial pilot sought voluntary participation from faculty, graduate students, and staff affiliated with the Department of Anatomy, Cell Biology, and Physiology and/or the graduate school at Indiana University School of Medicine. Two open information sessions were held describing the purpose and structure of *Forward Mentoring* and targeted announcements were made at faculty meetings and student/staff gatherings. Participants were offered $100 compensation upon program completion. All proposed studies were performed with Institutional Review Board (IRB) approval and all participants provided written informed consent.

#### Matching Process

An intentional pair selection process was utilized to assign mentoring dyads. A matching questionnaire was created to learn about participants’ career stage, identity, skills, and motivations, and to understand what participants hoped to gain through participation in the program. We then used the questionnaire to guide pair matching—aiming to match those with similar motivations/goals but differing in lived experiences and exposure to professional skills development.

### Goals

Below are the goals for *Forward Mentoring*:

1. Broaden perspectives and increase sense of belonging/connectedness through building relationships (connect).
2. Enhance leadership capacity and cultural humility through open, honest discussions (grow).
3. Increase confidence and self-efficacy through practical skill application and reflection (reflect).

### Conceptual Framework

**Fig. 1:**
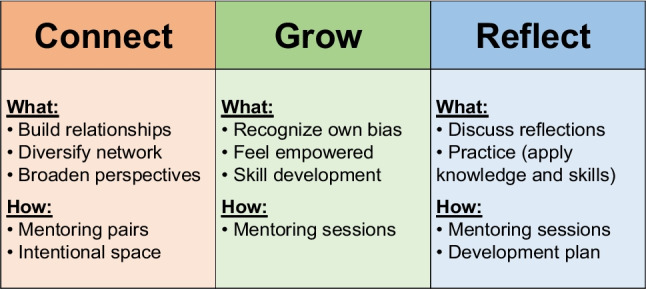
Conceptual Framework for *Forward Mentoring*. *Connect*: Increase sense of belonging and connectedness and broaden perspectives through building meaningful relationships. *Grow*: Gain awareness of self and others, and improve cultural competence/humility, empathy, trust building, curiosity, communication, and feedback skills. *Reflect*: Discuss, practice, and reflect on new skill application

Program goals are achieved through applying a framework centered around connection, growth, and reflection (Fig. 1). This framework draws on self-directed and experiential learning theories by emphasizing relationships that foster growth through discussion, reflection, and practical skill application [21, 26]. Connection serves as a foundational programmatic element. The goals associated with this element are building relationships, diversifying networks, broadening perspectives, and facilitating a sense of belonging and inclusion through open conversations with individuals from different backgrounds and career stages. This is achieved through the creation of intentional spaces for mentors and mentees to ask questions, practice, make mistakes, tell stories, and share perspectives. The second element, grow, aligns with the goals of increased leadership capacity and cultural humility through improving self/other awareness, communication, trust building, feedback skills, and increasing empathy. Session resources and guides provide content, discussion-based prompts, and scenarios that promote learning, reflection, and practice within these domains. The final element, reflect, serves the goals of promoting confidence and self-efficacy along with an increased ability to apply skills and cultivate an environment that values and promotes inclusivity and belonging. Pre-program bias self-assessments, session guides, and final goal-setting activities contribute to mentor and mentee reflection. *Forward Mentoring* is structured into six reverse mentoring sessions designed to support growth in each of these areas.

### Programmatic Components

#### Orientation

After recruiting and matching pairs, mentors and mentees participated in a two hour program orientation session. Two of the *Forward Mentoring* directors facilitated the orientation, one junior faculty/staff and one senior faculty member. The orientation was created to review the program goals and structure, to introduce pairs, and to lay the foundation for brave spaces in the mentoring sessions. Indeed, a successful reverse mentoring relationship requires psychological safety, vulnerability, and trust [23]. Thus, in addition to program review, the orientation featured a container building activity, a discussion/activity around the *Forward Mentoring* agreement, and an inclusive mindset discussion using Ruchika Malhotra’s BRIDGE Framework [28].

#### Session Structure/Design

The core *Forward Mentoring* pilot experience consisted of six mentoring sessions, spaced out over 4 months. The themes/topics for the sessions were based on the overall goals of the program. Each session guide supported experiential learning through materials designed to promote connection, learning, discussion, and reflection. The guides began with a prompt that helped pairs connect (e.g., “What is something that brings joy or meaning into your life?”). Pairs were then asked to reflect on the previous session and the time since their last meeting. Next, some topic-based definitions, quotes, and/or examples were provided. The discussion section included a series of questions to promote conversation based on the session theme/topic. Finally, scenarios were provided for pairs to practice skills introduced in the session. The sessions ended with another opportunity for reflection and prompted pairs to set a date/time for their next meeting. Participants were invited to reach out to program facilitators at any time with questions or concerns. Additionally, program facilitators held an informal check-in halfway through the sessions for peer connection (senior faculty facilitator met with mentees and junior faculty faciliator met with mentors) and to provide support, answer questions, and collect and address feedback.

#### Session Topics

**Session I** **: Get to Know Each Other**

Goals: connection, build relationship, recognize own biases, build session foundation.

- Set goals for and design session success.
- Reflect on biases [3] and the power of storytelling [12].
- Discuss the BRIDGE framework from Ruchika Malhotra’s book *Inclusion on Purpose*, “BRIDGE: Be uncomfortable, Reflect on what you don’t know, Invite feedback, Don’t get defensive, Grow from your mistakes, Expect that change takes time” [28] (p. 42).

**Session II** **: Empathy and Perspective Taking**

Goals: connection and belonging, build empathy and perspective taking skills.

- Understand differences between sympathy, compassionate empathy, emotional empathy, cognitive empathy [4] [33].
- Consider the role of empathy in perspective taking [16].
- How do empathy and perspective taking show up (e.g., challenges, how to practice)?

**Session III** **: The Role of Curiosity in Building Trust**

Goals: cultivate curiosity, trust building skills.

- Reflect on the importance of curiosity in relationships [37].
- Identify challenges associated with building trust [19].
- Reflect on use of the phrase, “I don’t know,” how to approach with curiosity, and the role of curiosity in building trust.

**Session IV** **: Fostering Courageous Cultures Through Meaningful Connection**

Goals: identify, practice communication and connection skills: active listening, acknowledging/validating (empathy), and providing support.

- Discuss what a courageous culture looks like.
- Identify communication strategies for difficult/courageous conversations [5].
- Reflect on a scenario based on cultivating connections through active listening, acknowledgement, validating feelings and emotions, and providing support [6].

**Session V** **: Feedback: Moving Beyond the Transactional**

Goals: develop self-efficacy and practice communication and feedback skills.

- Discuss psychological safety, active listening, and trust [18].
- Learn about and reflect on go-to feedback style, preferences for receiving feedback, and practice ways to invite feedback [10, 36].

**Session VI** **: Reflection and Practical Application**

Goals: reflect on skills learned and how to carry them forward.

- Reflection prompts to help participants complete an action plan:
  o What have you learned and what will you carry forward?
  o How will you practice what you have learned?
  o Where do you want to aim your energy moving forward and why?

#### Evaluation Plan

The *Forward Mentoring* evaluation plan was created to assess programmatic impact on participant learning and behavior change and to identify improvements for future iterations of the program. Kirkpatrick’s model was used as a foundation to collect both qualitative and quantitative data to obtain a clear understanding of participant experiences [25]. Quantitative data was gathered through a mid-point and final survey. Qualitative data was collected through surveys, a final focus group, and a 3-month follow-up questionnaire. Survey, follow-up questionnaire, and focus group questions were designed to evaluate the impact of the reverse mentoring approach including program satisfaction/reaction, learning, growth, and behavior change using the following metrics based on the first three of four levels in Kirkpatrick’s model [25]:

1. Level 1: Reaction—measured participant engagement and satisfaction; relevancy to work.
2. Level 2: Learning—measured broadening of perspectives, self-awareness, skill development (e.g., communication, empathy), and personal growth.
3. Level 3: Behavior—skill application/behavior change (e.g., ability to build trust, approach with curiosity, give and receive feedback, self-efficacy).
4. Level 4: Results—not assessed in this pilot.

#### Surveys

*Forward Mentoring* surveys measured reactions, learning, and behavior change through Likert scale and open-ended questions. Participants were asked to rate the impact of the bias self-assessments and the effectiveness of the mentoring sessions on broadening perspectives, establishing relationships, developing trust, responding with empathy, increasing comfort in asking questions and sharing perspectives. Participants had space to report takeaways from the sessions, modalities that supported the takeaways, goals/reflections, and programmatic suggestions.

#### Focus Groups

Two focus groups were held, one for mentors and one for mentees. Focus groups were designed to evaluate participant satisfaction, growth/learning, and behavior change as well as programmatic quality and future improvements. Participants were asked open-ended questions to elicit feedback on the matching process, orientation, and reverse mentoring sessions, specifically regarding the structure, timing, length, themes/topics, and session guides. They were also invited to identify program strengths, impact, learning, skill development, and practical application based on the session themes. Finally, mentors and mentees were asked to provide recommendations for program improvement.

#### Three-Month Follow-Up Questionnaire

All participants were sent the same follow-up questionnaire 3 months after program completion. The questionnaire included three open-ended questions to assess the continual impact of programmatic learning and growth on behavior change. Questions included the following: What impact has the program had on you? What, if anything, are you doing differently as a result of participating in the program? What are your biggest takeaways from participating in the program?

## Results

**Fig. 2:**
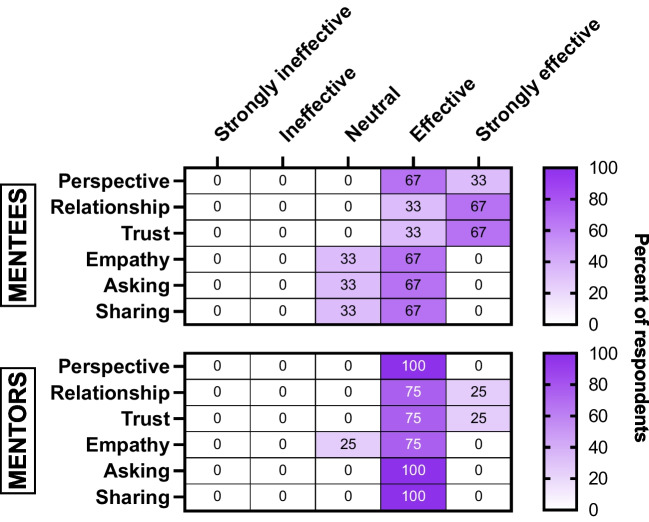
Selected final survey results. Shown are heatmaps summarizing mentee (top) and mentor (bottom) responses (*n* = 10) to prompts based on the question “How effective or ineffective were the mentoring sessions in doing the following? Full prompts: ‘Perspective’- Broadened your perspectives; ‘Relationship’- Established a relationship with your mentor/mentee; ‘Trust’- Developed a sense of trust with my mentor/mentee; ‘Empathy’- Increased ability to respond with empathy; ‘Asking’- Increased comfort in asking questions; ‘Sharing’- Increased comfort sharing your perspective.”

Overall, program participants were highly positive about the value and impact of the *Forward Mentoring* program. Survey, focus group, and follow-up questionnaire data suggested that the six-session experience yielded new connections, enhanced cultural humility via broadened perspectives and greater self-awareness, increased sense of belonging and empowerment, and supported skill development in the areas of empathy, curiosity, feedback, and navigating difficult situations (Fig. 2 and below).

Qualitative data was analyzed using an inductive approach to identify key patterns and themes that represent participants’ experiences. Data were categorized based on recurring patterns that emerged. A post analysis summary is provided to highlight participant experiences, and programmatic impact.

### Qualitative Findings

Key study findings include participant reflections on the range and progression of session topics, relationship building and connection, and the value of having an empowering and comfortable space to listen and be heard. Additional participant insights included perceived gains in awareness of self and others, personal and professional growth, and value and appreciation of the program.

Mentors and mentees expressed appreciation for the range, depth, and logical flow of themes in the session guides. One mentor stated, “I continued to notice that each topic built on the previous one. I especially liked that we started with recognizing our own biases.” Participants also emphasized the benefits of diving into topics not typically discussed in the workplace. Mentees noted, “We don’t see these important topics in other trainings” and “These are conversations that we would not otherwise have – talking about topics such as trust, and empathy are next level.” Participant engagement in meaningful and thought-provoking discussion about human-centered leadership skills highlights the innovative programmatic design of *Forward Mentoring*.

According to both mentees and mentors, the program fostered meaningful and lasting connection. Mentoring pairs expressed incredible value in forming new relationships and connecting across different backgrounds and career stages. Mentees noted, “You develop a relationship that is beyond superficial” and “We were able to develop relationships that we otherwise would not have – especially with mentors from different levels.” Additionally, mentors indicated deep and lasting impacts from the connections forged through the program stating, “I found it to be deeply impactful and a true connection between very disparate people” and “We really bonded very well so if I ever need anything I can go over there and ask for help and insights.” Building relationships [across differences] is essential for cultivating cultural competence and humility which, according to one mentee, “allows me to foster a more collaborative and positive work environment.” Creating new connections as a result of program participation has had a lasting impact on workplace relationships.

Participants highlighted how the sessions provided a level playing field and a space to listen and be heard. One mentee expressed that the sessions provided a comfortable space where “we could talk about everything.” Another mentee reflected that it is rare, in a hierarchial system, to have these types of conversations with others that are in different career stages, noting “these types of converstations just don’t happen.” Further, the participants indicated that the intentionality of the sessions fostered an environment of trust and mutual respect which empowered participants to express their opinions and engage in deep discourse around meaningful topics. One mentor stated, “It really gave me the power to be able to express how I feel” and “I felt that my voice was listened to during these sessions.” Ultimately, mentor and mentees expressed that the open and collaborative environments cultivated during the sessions allowed them to express their viewpoints, listen intently, and understand others’ perspectives.

Engaging in the mentoring process further supported participants in building self and other awareness. The program design invited participants to recognize their own biases and encouraged discussion with their partners. As a result, one mentor remarked that the program “has provided me with the tools to recognize [my] biases and prejudices, the language to discuss them, and the knowledge of how to mitigate them as best we can in our personal and professional lives.” Mentees reported exposure to different perspectives, enhanced introspection, and increased thoughtfulness in interactions with others which “changed the way I [will] interact with incoming trainees.” Similarly, a mentor noted that the increased awareness of how differing lived experiences impact behavior and beliefs impacts “the way I interact with others in a work environment.” Another mentor stated, “I noticed I am paying more attention when I talk to others.” Engaging in these reciprocal relationships supported mentors and mentees in understanding themselves which improved their ability to empathize and consider how they will interact more mindfully with others in the future.

Program participants shared a variety of insights that indicated personal and professional growth in cultural humility and human-centered leadership skills. Mentees and mentors reported practicing active listening, empathy, giving and receiving feedback, increased thoughtfulness when interacting with others, and improved relationships and mentoring capabilities. One mentee summarized their program takeaways as “practicing curiosity to build trust,” “I learned how I could do it [provide feedback] constructively,” and “It revealed the importance of psychological safety and connection.” Reflecting on skill development/program participation, one mentor reported learning “the importance of active listening and empathy in professional settings” and “how crucial it is to fully understand and appreciate others’ viewpoints to build stronger, more collaborative relationships.” When considering behavior change, one mentee said they are now “taking more time to check-in and learn about and address trainee needs to foster growth.” Another mentee reflected that they realized how important “a feeling of belonging can be to someone’s satisfaction” at work.

Overall, the qualitative data suggests that *Forward Mentoring* contributed to introspection, broadened perspectives, and meaningful connections, while increasing participants’ ability to cultivate inclusive and comfortable spaces, as well as navigate complex interpersonal dynamics. One mentor summarized the importance of creating intentional spaces and building connections when they said, “it is not enough to hear others, but to listen with intent to lift their voices and work toward a more inclusive future.” Beyond learning and growth, mentees, in particular, expressed their appreciation for the program stating, “this was excellent,” an “incredible value,” and it “provided a path for me to be a better mentor.” Ultimately, mentors and mentees attributed program success to authentic/genuine connection, supportives spaces, and thoughtful session guides that encouraged participants to engage in intentional conversations around on deep, meaningful, human-centered leadership topics. As one mentee put succinctly, “This is a very useful and well-designed program. I recommend it for everyone: trainees, staff and mentors.” These and all qualitative data are summarized in Table 1.

**Table 1:** Summary of qualitative data

Finding	Evidence
Logical progression and range of topics	“I continued to notice that each topic built on the previous one. I especially liked that we started with recognizing our own biases.” (mentor) “The guides were great and very helpful in making every session interesting and productive.” (mentor) “Being able to speak to somebody that I usually look at as a superior or mentor for myself—it was a great opportunity to be able to sit across from them face to face and have these active and honest conversations, and we were able to talk about a lot of sensitive topics that I feel like before, we never would have talked about.” (mentor) “These are conversations that we would not otherwise have – talking about topics such as trust, and empathy are next level.” (mentee) “We don’t see these important topics in other trainings.” (mentee)
Relationship building and connection	“I found it to be deeply impactful and a true connection between very disparate people.” (mentor) “I think making that connection was really impactful for both of us.” (mentee) “You develop a relationship that is beyond superficial.” (mentee) “We really bonded very well so if I ever need anything I can over there and ask for help and insights.” (mentor) “We were able to develop relationships that we otherwise would not have – especially with mentors from different levels.” (mentee) Improved interactions and relationships “allow me to foster a more collaborative and positive work environment.” (mentee)
Space to listen and be heard	“Having the space where two parties are just comfortable to talk about everything.” (mentee) “It really gave me the power to be able to express how I feel and express my views on certain topics, and then, of course, listen to theirs and be able to find common ground in a lot of situations. So, it was really empowering in that sense. So, I say, one of the strengths is the way that I felt that my voice was listened to during these sessions.” (mentor) “Taking the time to listen to people more and understand their perspective.” (mentor) “These types of conversations don’t just happen.” (mentee)
Self and other awareness	“I noticed that I’m paying more attention when I talk to others.” (mentor) “I definitely appreciated the reverse mentoring program, and I have found it extremely informative. It has provided me with the tools to recognize [my] biases and prejudices, the language to discuss them, and the knowledge of how to mitigate them as best we can in our personal and professional lives.” (mentor) Increased thoughtfulness in interactions with others and “changed the way I interact with incoming trainees.” (mentee) “Hearing some of their viewpoints on the way they approach things was interesting and different and definitely not how I thought about it. So, it was a good experience to get a different perspective on the way that we approach or think about some of these different types of topics.” (mentee) Increased awareness of how differing lived experiences impact behavior and beliefs impacts “the way I interact with others in a work environment.” “It’s made me more introspective about some things and more considerate about the way I’m approaching certain situations, or plan to with future trainees.” (mentor)
Personal and professional insights and growth	“The importance of active listening and empathy in professional settings. I’ve learned how crucial it is to fully understand and appreciate others’ viewpoints to build stronger, more collaborative relationships.” (mentor) “It is not enough to hear others, but to listen with intent to lift their voices and work towards a more inclusive future.” (mentor) Session takeaways: “Practicing curiosity to build trust.”; “I learned how I could do it [provide feedback] constructively”; “It revealed the importance of psychological safety and connection.” (mentee) “With all the resources that were given, I feel like it has made me think of what kind of mentor I want to be in the future.” (mentor) “Taking more time to check-in and learn about and address trainee needs and foster growth.” (mentee) Realized the importance “a feeling of belonging can be to someone’s satisfaction.” (mentee)
Value and appreciation of the program	“Provided a path for me to be a better mentor.” (mentee) “This was excellent.” “Incredible value.” (mentee) “This program is really special. I met with my mentor this week and we had such a rich conversation. I’m so glad you built this.” (mentee) “This is a very useful and well-designed program. I recommend it for everyone, trainees, staff, and mentors” (mentee)

## Discussion

Without practices that actively foster inclusivity, academic research environments will fail to recruit and retain diverse talent—thus threatening innovation, growth, and progress. While many such activities exist (at Indiana University and beyond), few challenge hierarchical structures that impose significant barriers to inclusivity. While initial *Forward Mentoring* program rationale began as a response to departmental Diversity Engagement Survey (DES) survey [34] results indicating low scores in the areas of cultural competence and sense of belonging, the program purpose evolved into a broader desire to respond to overall culture and climate concerns that exist in academic science and impact the retention of historically underrepresented faculty. IUSM is not immune to these issues and Indiana University has related core principles in their 2030 plan including (1) a commitment to diversity, equity, and inclusion and (2) cultivating a culture of respect and integrity. These align with the *Forward Mentoring* framework which centers connection, growth, and reflection with the aim of cultivating more welcoming and inclusive environments. *Forward Mentoring*, addresses a gap in developmental programming by leaning into the diverse experiences of trainees/junior colleagues, amplifying and celebrating their perspectives, and seeding open dialogue between present and future leaders. Initial feedback from this *Forward Mentoring* pilot was highly positive, with clear benefits to both mentors and mentees in terms of personal growth, self-awareness, perspective shifting, empathy capacity, and feedback skills.

### Limitations

*Forward Mentoring* is not without limitations. First, we acknowledge that this was a small pilot of ten individuals, most of whom are from the same department. Thus, our ability to interpret and extrapolate these findings is limited. Second, though immediate feedback was positive, we do not yet have a sense of the long-term impact *Forward Mentoring* will have on program participants. In that regard, a 12-month post-program follow-up is planned that will query self-reported behavior change and/or perduring perspective shifts. Third, we emphasized (and prioritized) willing and active engagement, and thus recruited individuals who were already on a committed path of personal growth. Considering that many do not have the capacity to make such commitments, it remains questionable whether *Forward Mentoring* (or any such intervention that requires significant buy-in) can significantly “move the needle” with respect to broader institutional culture change. Compulsory participation would likely jeopardize the fragile balance of power that *Forward Mentoring* seeks to cultivate, though perhaps incentives (e.g., time or resources) could help broaden engagement by reducing barriers to participation.

### Future Implications

Despite these limitations, *Forward Mentoring* has important implications for professional development in the higher education sphere. Indeed, the ripple effect of *Forward Mentoring* is significant as it uniquely amplifies the voices and experiences of trainees/those with little institutional influence and brings those perspectives to leadership tables via relationships/personal connections. Enriching leadership conversations with diverse perspectives will likely lead to more informed and socially conscious decision-making and has the potential to positively impact institutional cultures by embracing and prioritizing inclusive, inviting, and welcoming spaces.

Given the overwhelmingly positive feedback of the pilot, rapid expansion may be tempting to reach a broader audience. We cautiously agree while contending that program growth must be considered with intention and care in order to maintain programmatic benefits and impact. For example, one logical way to expand *Forward Mentoring* may be to open it up to the wider Indiana University community, while maintaining central program administration within the Indiana University School of Medicine. In this model, regional leaders would support local implementation and connection building. This type of expansion would entail virtual and in-person components and would introduce physical distance as an unknown variable. Careful monitoring of the quality of the orientation session, ability of facilitators to support mentoring pairs, and relationship building between mentors and mentees could make this possible. Similarly, future iterations of *Forward Mentoring* could include cross-institutional or cross-departmental mentor/mentee pairing to broaden reach and maximize differences between pairs, while maintaining a small mentor/mentee cohort to ensure that program facilitators can provide adequate support throughout the reverse mentoring process. We do not yet know if the potential for greater participation, wider reach, and increased ability to maximize differences between pairs would outweigh the benefits of fully in-person, locally based sessions.

As should be the case when introducing new variables into an existing ecosystem, contextual assessment of other related developmental offerings would be prudent. Beyond serving as a stand-alone program, *Forward Mentoring* has the potential to enrich or enhance pre-existing professional development programs. For example, *Forward Mentoring* could be part of a menu of experiential “reinforcement” opportunities to supplement other developmental activities, including anti-bias training modules, mentoring workshops, or leadership trainings. Providing options for hands-on professional development opportunities gives participants autonomy, supports individual motivation, and can enhance skill retention. Further, offering reverse mentoring as a choice/experiential opportunity tied to existing programming may further encourage participation and buy-in for those who may be reluctant to pursue reverse mentoring on its own.

From a practical standpoint, facilitator composition and dynamics, program size, and administrative support should also be thoughtfully considered alongside program expansion efforts for the following reasons: First, we observed that a mixed leadership/facilitator team was helpful for participants in that targeted (and sometimes separate) conversations during orientation and check-ins seemed to support unique mentor/mentee needs. Second, the small size of the *Forward Mentoring* pilot allowed mentors and mentees to build relationships with program facilitators during orientation and subsequent informal check-ins. This setup created a supportive environment for participants to be able to reach out to program facilitators with any questions or concerns throughout the process. Third, it is essential that program team members have ample time to dedicate to planning, creating, and adapting reverse mentoring session resources and guides in response to participant feedback and needs throughout the program. Having a facilitation team that cares about the program participants and their experiences throughout the program is key to a successful reverse mentoring program. Intentional prioritization of these key success factors, along with slow growth that maintains space for supportive and meaningful connections, will help to ensure that program benefits are not lost as *Forward Mentoring* grows.

## Conclusion

We conclude that programs such as *Forward Mentoring* have the potential to contribute to cultural transformation within institutes of higher education. Indeed, “…telling our stories and reflecting on our experiences more transparently – by including the heart and hands as well as the brain – is essential to understanding what we bring to the teaching and learning endeavor” [20] (p. 194). This is what *Forward Mentoring* seeks to do—to create an intentional space to bring people from different backgrounds together (individuals who might not otherwise connect organically) to share their stories and perspectives. By doing this, we believe that programs such as *Forward Mentoring* will contribute to higher education teaching, mentoring, research, and learning environments that are more culturally competent, empowered, curious, connected, innovative, productive, empathetic, and inclusive.
